# SARS-COV-2 as potential microRNA sponge in COVID-19 patients

**DOI:** 10.1186/s12920-022-01243-7

**Published:** 2022-04-23

**Authors:** Chang Li, Rebecca Wang, Aurora Wu, Tina Yuan, Kevin Song, Yongsheng Bai, Xiaoming Liu

**Affiliations:** 1 USF Genomics and College of Public Health, University of South Florida, Tampa, FL USA; 2 Pioneer High School, Ann Arbor, MI USA; 3 Emma Willard School, Troy, NY USA; 4 The Roeper School, Birmingham, MI USA; 5 Credit Suisse, New York, NY USA; 6 Next-Gen Intelligent Science Training, Ann Arbor, MI USA; 7 Department of Biology, Eastern Michigan University, Ypsilanti, MI 48197 USA

**Keywords:** MicroRNA, SARS-CoV-2, Viral infection, COVID-19, MicroRNA target, ACE2

## Abstract

**Background:**

MicroRNAs (miRNAs) are a class of small non-coding RNA that can downregulate their targets by selectively binding to the 3′ untranslated region (3′UTR) of most messenger RNAs (mRNAs) in the human genome. MiRNAs can interact with other molecules such as viruses and act as a mediator for viral infection. In this study, we examined whether, and to what extent, the SARS-CoV-2 virus can serve as a “sponge” for human miRNAs.

**Results:**

We identified multiple potential miRNA/target pairs that may be disrupted during SARS-CoV-2 infection. Using miRNA expression profiles and RNA-seq from published studies, we further identified a highly confident list of 5 miRNA/target pairs that could be disrupted by the virus’s miRNA sponge effect, namely hsa-miR-374a-5p/APOL6, hsa-let-7f-1-3p/EIF4A2, hsa-miR-374a-3p/PARP11, hsa-miR-548d-3p/PSMA2 and hsa-miR-23b-3p/ZNFX1 pairs. Using single-cell RNA-sequencing based data, we identified two important miRNAs, hsa-miR-302c-5p and hsa-miR-16-5p, to be potential virus targeting miRNAs across multiple cell types from bronchoalveolar lavage fluid samples. We further validated some of our findings using miRNA and gene enrichment analyses and the results confirmed with findings from previous studies that some of these identified miRNA/target pairs are involved in ACE2 receptor network, regulating pro-inflammatory cytokines and in immune cell maturation and differentiation.

**Conclusion:**

Using publicly available databases and patient-related expression data, we found that acting as a “miRNA sponge” could be one explanation for SARS-CoV-2-mediated pathophysiological changes. This study provides a novel way of utilizing SARS-CoV-2 related data, with bioinformatics approaches, to help us better understand the etiology of the disease and its differential manifestation across individuals.

**Supplementary Information:**

The online version contains supplementary material available at 10.1186/s12920-022-01243-7.

## Background

During the past year, the coronavirus disease 2019 (COVID-19) as a major global pandemic has taken the lives of more than 2 million people. The highly transmissible virus, severe acute respiratory syndrome coronavirus 2 (SARS-CoV-2), is responsible for the world-wide spread of COVID-19. It is a single-strand, positive-sense RNA virus which belongs to betacoronavirus genera together with SARS-CoV and MERS-CoV (Middle East respiratory syndrome coronavirus) [1]. All of these viruses can cause severe respiratory symptoms such as the Acute Respiratory Distress Syndrome (ARDS) in human [2]. To overcome this public health crisis, numerous efforts have been made to try to understand the disease and guide the development of preventive and treatment strategies [3, 4], however the underlying molecular mechanism of COVID-19 pathogenesis is still not fully understood.

MicroRNAs (miRNA) are small (21–22 bp) noncoding RNAs that can selectively repress the expression of target mRNA(s) through binding to targets’ 3′ untranslated regions (UTRs) [5]. Human miRNAs not only can post-transcriptionally regulate mRNAs, but they can also interact with other single-strand RNAs such as viral genomes [6–8]. Multiple miRNAs have been reported to have either antiviral or disease-causing effects [9]. For example, hsa-miR-1-3p has been associated with inhibition of H3N2 virus replication [10] whereas miR-122 has been reported to stabilize viral genomes and facilitate viral replication in Hepatitis C virus [11]. Previous studies have investigated the possible effects of host miRNAs to act as anti- or pro- viral molecules to modify viral duplication among COVID-19 patients [9, 12]. However, few studies to date have investigated the possible sponge effect of miRNA-viral genome interactions to disrupt the regulatory network of host miRNAs [13, 14]. Such miRNA sponges can competitively sponge host miRNAs to deplete specific miRNAs, thereby disrupting normal pathways regulated by these miRNAs. As demonstrated by previous studies on other viruses such as Epstein-Barr virus [15], the miRNA sponge effect of these viruses is greatly associated with their malignance. This trend was also observed from a recent study which adopted in-silico tools to investigate the predicted miRNA targets across different strains of coronaviruses [13]. The authors observed a positive correlation between the number of miRNA target sites and the pathogenicity of the strains. Even though some primary evidence has shown that viruses can impact their hosts through acting as miRNA sponges, it is still not clear to what extent the SARS-CoV-2 virus can undertake similar mechanisms.

In this study, we utilized publicly available databases, including RNA-sequencing (RNA-seq) data and single-cell RNA-sequencing (scRNA-seq) data of COVID-19 patients and healthy controls, and multiple web-services to explore the potential role of SARS-CoV-2 as a miRNA sponge. The process identified multiple candidate miRNA-gene pairs that are likely affected through SARS-CoV-2's miRNA sponge mechanism. Our study can shed new light on the pathogenesis of SARS-CoV-2 infection through the exploration of its potential role as a miRNA sponge.

## Results and discussion

### MiRNA families that interact with SARS-CoV-2 genome

**Fig. 1:**
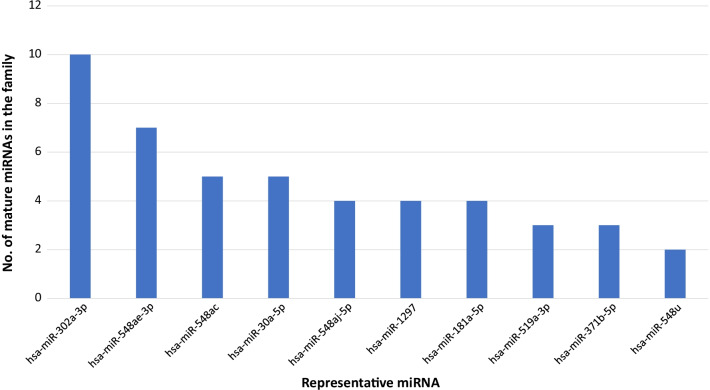
Top 10 miRNA families that are predicted to interact with SARS-CoV-2 genome. Only representative mature miRNA names are shown

Nine hundred potential viruses targeting miRNAs (VTMs) for the SARS-CoV-2 genome passed the score filter (target score ≥ 50) and 91 miRNAs were categorized into high-confidence VTMs (target score ≥ 90). We identified 24 miRNA families that were over-representated in these VTMs (Additional file 1). Top 10 most over-represented miRNA families were shown in Fig. 1. The family with most number of VTMs was the miR-302-3p/372-3p/373-3p/520-3p family, which had 10 VTMs and a seed sequence of AAGUGCU. Members from this family have been reported to act as antiviral miRNAs against multiple virus infections [32]. Interestingly, a recent study has reported that this miRNA family can target multiple pro-inflammatory cytokines such as IL-6 and IL-8 [33]. The activation of these pro-inflammatory genes can lead to a "cytokine storm" which may then cause the signature ARDS observed in severe COVID-19 cases. Another miRNA family that was previously reported, the miR-30a-5p family, has showed decreased expression due to an alphacoronavirus infection which led to an upregulated SOCS1 gene (suppressor of cytokine signaling protein 1) expression [34]. These observations support the miRNA sponge effect of SARS-CoV-2 and it can provide a viable explantation for the activation of these genes through decreased levels of their regulating miRNAs. These proposed miRNA-gene axes could then lead to hyperinflammation in different COVID-19 patients. Moreover, a recent study reported that hsa-miR-548d-3p, a member from the miR-548ac family, showed decreased expression in COVID-19 patients [25]. These evidence showed that miRNAs that can target SARS-CoV-2 or other coronaviruses could potentially activate pro-inflammatory cytokines and cause a deep inflammatory state for the patient. Even though some other miRNA families we identified here have not been reported previously such the miR-1297 family, we expect that they could contribute to the severity of COVID-19 through these known or other novel biological pathways.

### VTMs are enriched in candidate differentially expressed genes

We further examined if the targets of the high-confidence VTMs are significantly enriched in our candidate gene list (upregulated genes) compared to our background gene list (downregulated genes). Using experimentally validated miRNA-target pairs from miRTarBase, we observed 59 VTMs out of the 373 potential miRNAs that can target genes from candidate gene list and only 28 VTMs out of 330 miRNAs that can target genes from background gene list. Even though the two lists have similar number of targeting miRNAs (n = 373, n = 330), our candidate gene list which contained genes that are upregulated in COVID-19 patients showed significantly more binding VTMs (chi-square *p* value < 0.001). This highlighted that these mild-to-moderately upregulated genes in our candidate gene list are more likely to be regulated by VTMs. With the presence of SARS-CoV-2, these VTMs that can simultaneously target the virus genome and regulate human genes are likely to have insufficient expression to maintain their normal functionalities. This could result in upregulation of their target genes and would lead to downstream perturbation of normal cellular functions. This miRNA sponge effect is a plausible explanation for the observed phenomenon based on patient RNA-seq data, a high quality in-silico miRNA prediction tool and experimentally validated miRNA-gene interactions in human. The complete list of VTM-gene pairs that are potentially affected by miRNA sponge effect are available in Additional file 2.

### MiRNA-gene-pathway enrichment analysis

With VTM-gene pairs identified from previous step, we investigated if any of these VTMs are enriched in their targeted genes. Using miEAA web-service, we found that these VTMs are significantly enriched in 885 genes (FDR adjusted *p* value < 0.01; Additional file 3). We further checked if any of these genes are in the 26 genes that are differentially expressed in our patient data. After this filtering step, 6 genes showed both significant enrichment in their targeting VTMs and were significantly upregulated in COVID-19 patients (Table 1). These 6 genes were observed to be simultaneously regulated by multiple VTMs, whereas the other 20 genes are more likely to be regulated by a small number of VTMs. Thus, this list of 6 genes could be more heavily affected by the miRNA sponge effect of SARS-CoV-2 virus.

**Table 1:** Differentially expressed genes with enriched VTMs

Target gene	*p* value	FDR adjusted *p* value	Expected No. of VTMs	Observed No. of VTMs
PSMA2	1.49 × 10^–32^	1.58 × 10^–28^	2.46	31
ZNFX1	2.21 × 10^–21^	7.83 × 10^–18^	4.17	31
APOL6	1.63 × 10^–17^	2.17 × 10^–14^	8.54	39
RABGAP1L	3.97 × 10^–14^	1.51 × 10^–11^	5.87	29
EIF4A2	2.71 × 10^–06^	6.67 × 10^–05^	2.94	13
PARP11	3.44 × 10^–05^	5.45 × 10^–04^	2.66	11

Using the miEAA and miRWalk, we also examined the enriched pathways of genes that are targeted by the VTMs (Additional file 4). Multiple pathways were identified to be regulated by VTMs and their targeted genes, such as cell apoptosis pathway, the TGF-beta signaling pathway and the aforementioned cytokine signal pathways (IL-1, IL-6). While cell apoptosis pathway and cytokin signal pathways were mechanistically associated with SARS-CoV-2 infection, multiple papers have discussed the potential of COVID-19 treatment targeting TGF-beta pathway [35, 36]. These supporting evidence accentuated the validity of our approach in identifying biologically relevant and actionable pathways through miRNA related enrichment analyses. Moreoever, since there still lacks specific and effective treament strategies for COVID-19, the miRNA-gene-pathway axes we identified here could provide a novel perspective on the pathogenesis of the disease which may lead to identification and development of potential treatment strategies.

### Candidate VTM-gene pairs may have opposite roles during SARS-CoV-2 infection

Given the previously reported anti-viral function of host miRNAs, we expect that candidate VTM-gene pairs we identified to have opposing roles in contributing to viral disease pathophysiology. The first role is in disrupting gene expression regulators caused by the miRNA sponge effect of the invading virus. The second role is their antiviral role in repressing viral replication. Thus, we hypothesize that these potential miRNA sponge effects could have opposite influences on viral infection and disease progression. They can facilitate disease progression by disrupting host regulating miRNAs by sponging host miRNAs. At the same time, they may be able to inhibit viral replication by sponging antiviral miRNAs to SARS-CoV-2 genome. To explore this hypothesis, we calculated Spearman's rank correlation between the expression of our 26 candidate genes and the viral count in patients (Table 2). We found that 2 of these genes showed significant negative correlation with viral counts, namely EIF4A2 and PRMT7. VTMs associated with these 2 genes are likely to play two roles in SARS-CoV-2 infection: antiviral miRNAs and sponged/disrupted miRNAs affected by the virus. Since viral load can be considered as a surrogate for disease severity [37], this observation also implies the possible contribution of miRNA sponge mechanism to the severity of disease. The other 24 genes showed either non-significant negative correlation or positive correlation with viral count, which indicates that their associations with diease severity are not directly related to viral count but through other mechanisms such as miRNA sponge.

**Table 2:** Candidate gene expressions and their correlation with viral count among COVID-19 patients

Gene symbol	Correlation	*p* value	FDR adjusted *p* value
EIF4A2*	− 0.37	8.76 × 10^–4^	0.02
PRMT7*	− 0.34	2.18 × 10^–3^	0.03
PSMA6	− 0.29	0.01	0.09
AGRN	− 0.24	0.03	0.17
IRF9	− 0.24	0.04	0.17
RABGAP1L	− 0.23	0.04	0.17
PSMA2	− 0.22	0.05	0.17
CMTR1	− 0.22	0.05	0.17
JADE2	− 0.20	0.08	0.23
PARP11	− 0.19	0.10	0.27
PSMB8	− 0.17	0.14	0.32
GBP3	− 0.16	0.18	0.38
APOL6	− 0.14	0.22	0.42
TRIM14	− 0.14	0.23	0.42
RBCK1	− 0.12	0.29	0.50
PARP10	− 0.09	0.44	0.68
PNPT1	− 0.08	0.48	0.68
TRAFD1	− 0.08	0.48	0.68
TDRD7	− 0.08	0.50	0.68
TGM2	− 0.07	0.55	0.71
NUB1	0.04	0.76	0.82
TMSB10	0.03	0.77	0.82
OPTN	− 0.03	0.78	0.82
CNP	− 0.03	0.79	0.82
SLC25A28	0.03	0.79	0.82
ZNFX1	− 0.02	0.88	0.88

*Significant negative correlation between gene expression and viral count

### Differentially expressed miRNAs and their targets in SARS-CoV-2 infected cells

To pinpoint which VTMs are more likely affected by the SARS-CoV-2’s miRNA sponge effect, we obtained 44 significantly upregulated or downregulated miRNAs between SARS-CoV-2 infected and control Calu-3 cells from an independent study [25]. Among these differentially expressed miRNAs, 28 of them were downregulated and 16 of them were upregulated after infected by SARS-CoV-2 virus. Among the 16 upregulated miRNAs, only 2 of them were present in our candidate VTMs. Among the 28 downregulated miRNAs, 15 of them were in our candidate VTM list and 5 of them were experimentally validated to be able to target the 5 genes from our candidate gene list (Table 3). As expected, our identified VTMs are significantly enriched in the list of downregulated miRNAs (*p* value < 0.01). Additionally, these 5 validated genes were also identified in our previous analyses. Based on these multiple lines of evidence, namely gene upregulation, miRNA downregulation, and our bioinformatics investigations, these five VTM-gene pairs are highly likely to be affected by the SARS-CoV-2 virus through miRNA sponge effect.

**Table 3:** High confidence VTM-gene pairs supported by multiple lines of evidence

Gene symbol	Mature miRNA
APOL6	hsa-miR-374a-5p
EIF4A2	hsa-let-7f-1-3p
PARP11	hsa-miR-374a-3p
PSMA2	hsa-miR-548d-3p
ZNFX1	hsa-miR-23b-3p

Additionally, we identified the differentially expressed genes between COVID-19 patients and patients with other upper airway virus infections [38]. Among the 5 candidate VTM-gene pairs, only EIF4A2 gene showed significant upregulation in COVID-19 patients. This indicated that genes ZNFX1, PSMA2, APOL6 and PARP11 could be specifically involved in SARS-CoV-2 infection. A literature search was performed to find evidence of the association between the 5 candidate VTM-gene pairs and SARS-CoV-2 infection. Interestingly, the PSMA2 gene was previously identified as a hub gene that was upregulated in COVID-19 patients [39]. While the original study did not report any miRNA level evidence, we expect that hsa-miR-548d-3p that was sponged by the virus could be responsible for the upregulation of its target PSMA2 gene. Additionally, the ZNFX1 gene was reported in a study to be upregulated among severe COVID-19 cases who had asthma [13]. The similar upregulation was observed for the PARP11 gene in deceased COVID-19 patients' lung samples [40]. All these upregulated genes observed in COVID-19 patients from previous independent studies support our hypothesis of the miRNA sponge role of the SARS-CoV-2 virus. Even though only a few studies have focused on miRNA-gene relationships in COVID-19 patients, we expect that with our vigorous filtering steps and supportive evidence from previous studies, these five candidate VTM-gene pairs could be a promising starting point for future validations.

### Single-cell RNA-seq analysis identified cell-type specific miRNA sponge related VTM-gene pairs

Lastly, we applied our analysis pipeline to thirteen single-cell RNA-sequencing (scRNA-seq) samples from bronchoalveolar lavage fluid (BALF) [30]. Following the data pre-processing, integration and clustering steps proposed in the original paper, we identified the same number of cells as the original paper (n = 66,452). Using cell annotations from the paper's GitHub page (https://github.com/zhangzlab/covid_balf), we annotated all major cell types including epithelial cells, macrophages, neutrophils, myeloid dendritic cells (mDC), plasmacytoid dendritic cells (pDC), mast cells, natural killer (NK) cells, T and B cells. Next, cell-type specific analyses of DEGs were performed bewteen severe COVID-19 patients and moderate COVID-19 patients to examine if miRNA sponge effect could act as an explanation for severe COVID-19 cases. Four of the cell types showed DEGs that passed our fold-change filters, namely B, macrophages, mDCs and T cells (Additional file 5). We found out that hsa-miR-302c-5p can potentially affect genes from 3 out of the 4 cell types, including B cells, macrophages and mDCs. Interestingly, this miRNA has been reported to be a key regulator of ACE2 (angiotensin-conveting enzyme 2) network, the most important receptor for SARS-CoV-2 infection [41]. Therefore, the associated miRNA, hsa-miR-302c-5p, that is expected to be sponged by the SARS-CoV-2 genome, can potentially lead to increased ACE2 expression which has already been reporeted to be associated with severe COVID-19 cases [42]. Moreover, hsa-miR-16-5p is the miRNA that can target the most number of DEGs in macrophages (n = 15) and T cells (n = 10). This miRNA is another key regulator of the ACE2 network [41]. Additionally, deficiency of hsa-miR-16-5p has previously been reported to affect T cells’ cell cycle, survival, and differentiation [43]. These observations indicated the possible role of SARS-CoV-2 as a miRNA sponge and provide new insights into the possible disease mechanisms of severe COVID-19 through possible miRNAs-ACE2/immune cells-disease severity axes.

## Conclusion

In this study, we suggest that the SARS-CoV-2 virus could act as a miRNA sponge to disrupt the normal miRNA regulatory pathways. Based on multiple lines of evidence, we identified 5 high-confidence VTM-gene pairs that are most likely affected by miRNA sponge effect mediated by the SARS-CoV-2 virus. Some identified pathways such as the cytokine signaling pathway could be one explanation for ARDS and differential disease severity between COVID-19 patients. Additionally, we explored the possibility that miRNAs sponged by the virus could inhibit viral replication at the same time which complicated the functional role of SARS-CoV-2. Through the scRNA-seq analyses, we found out that hsa-miR-302c-5p and hsa-miR-16-5p could potentially affect SARS-CoV-2 infection through modulating ACE2 receptor related network.

Since our study and other currently available studies adopted bioinformatics approaches to investigate this issue, one key direction for future studies is to validate our findings including candidate VTM-gene pairs and affected biological pathways using experimental approaches. This can provide invaluable insights into the mechanism of COVID-19 and even other types of viral infections. Additionally, more patient data, especially data with greater heterogeneity, can be helpful in increasing the power to identify key and/or population specific regulatory pathways involved in the sponge mechanism. Further investigations of scRNA-seq data can potentially identify more cell-type specific or housekeeping VTMs. Other bioinformatics studies may use the miRNAs and especially VTMs to design and develop miRNA markers to perform patient risk assessment. In summary, we expect findings reported in our study could provide a valuable starting point for future experimental and functional validations to help us better understand and fight against COVID-19.

## Methods

### Retrieving reference genome of SARS-CoV-2 and miRNA target predictions

**Fig. 2:**
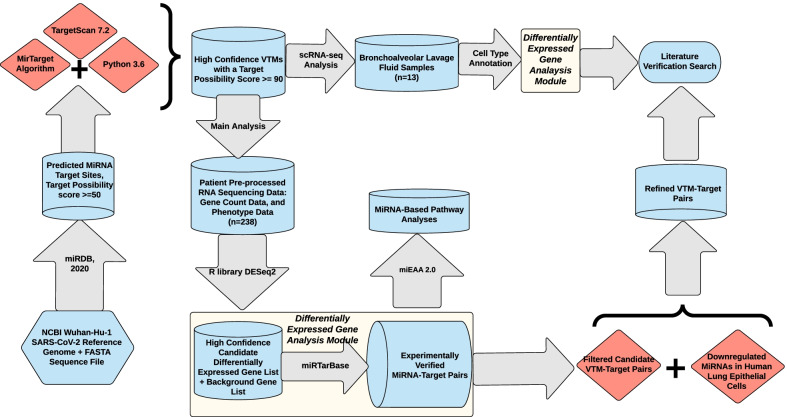
A brief summarization of the study workflow

A brief summarization of the workflow for this study was shown in Fig. 2. To identify candidate miRNAs that can pair with SARS-CoV-2, we first retrieved its reference genome, documented at NCBI. We used the Wuhan-Hu-1 genome as our reference genome for SARS-CoV-2, and the FASTA sequence file was retrieved from https://www.ncbi.nlm.nih.gov/nuccore/mn908947.3.

We used the custom prediction function from miRDB (http://mirdb.org) to predict potential miRNA target sites in the SARS-CoV-2 genome [16]. The SARS-CoV-2 reference genome was then submitted to the miRDB as a mRNA sequence, and the result was downloaded and further processed using a custom Python script. The miRDB webservice uses MirTarget as its prediction algorithm, which returns a quantitative measurement for each predicted target, indicating its likelihood of being a true targeting miRNA. For any potential targeting miRNA, miRDB returns a value between 50 and 100. All miRNAs with scores greater than or equal to 50 were used as potential virus targeting miRNAs (VTMs). MiRNAs with scores greater than or equal to 90, which correspond to the top 10% most confident predictions in our study, were kept as potential high-confidence VTMs. MiRNA family information was retrieved from TargetScanHuman at http://www.targetscan.org/cgi-bin/targetscan/data_download.vert72.cgi [17, 18].

### Retrieving COVID-19 patient data

The publicly available patient data were reported in a previous study [19]. Briefly, the data include patients that have been tested positive for SARS-CoV-2 by nasopharyngeal/oropharyngeal (NP/OP) swab polymerase chain reaction (PCR) in China (n = 94), no-symptom controls (n = 103) and patients who tested negative for SARS-CoV-2 but with other respiratory virus infections (n = 41). All the genotype (pre-processed gene counts) and phenotype data (including virus status and viral counts) were retrieved from the corresponding GitHub page: https://github.com/czbiohub/covid19-transcriptomics-pathogenesis-diagnostics-results.

### Identification of differentially expressed genes

Differentially expressed genes (DEGs) between COVID-19 patients and no-symptom controls were identified using the gene count data and R package DESeq2 (https://bioconductor.org/packages/release/bioc/html/DESeq2.html) [20]. Based on our assumption that SARS-CoV-2 can serve as a miRNA sponge to downregulate miRNA expression in human cells, we limited our candidate genes to those that are upregulated in COVID-19 patients. Additionally, since miRNAs' impact on gene expression levels is usually mild to moderate, we further limited the expression change of candidate genes to be between 1 and 2 folds, or equivalently a log_2_ fold change between 0 and 1 (ratio between gene expression in cases over gene expression in controls) [17]. An FDR adjusted *p* value of less than 1 × 10^–6^ was used to obtain a high-confidence candidate gene list that was upregulated. Since our focus was mildly-to-moderately upregulated genes, we set a more stringent *p* value threshold to ensure the quality of the identified DEGs. For our background gene list, we selected genes with fold-change between 0.5 and 1, or equivalently a log_2_ fold change between -1 and 0, with the same *p* value cut-off to match their magnitude of change with our candidate gene list. The final transcript names identified from previous step were converted to HUGO Gene Nomenclature Committee (HGNC) gene symbols using R package biomaRt [21, 22]. DEGs between COVID-19 patients and patients with other viral infections were identified using the same approach.

### Retrieving experimentally validated miRNA targets

To identify high-confidence miRNA targets for our candiate gene list, we retrieved experimentally verified miRNA-target pairs from miRTarBase (http://mirtarbase.cuhk.edu.cn/php/index.php) [23, 24] which were curated using natural language processing and manual surveys. Using a custom Python script, we kept only those miRNA-target pairs that involve genes in our candidate gene list and background gene list.

### Identification of enriched miRNAs that can target differentially expressed genes

To check if VTMs are enriched in our candidate gene list compared to the background gene list, we performed enrichment anlysis for the number of VTMs in each of these lists. Specifically, using the miRNA-target pairs reported in miRTarBase, the number of VTM-target pairs in our candidate gene list that are reported in miRTarBase (complete VTM-target pairs) and the number of VTM-target pairs in our background gene list that are reported in miRTarBase (background VTM-target pairs) were compared. Chi-squred test statistics was used to claim test significance.

To check which of our candidate VTMs are more likely to be affected by miRNA sponge mechanism, we retrieved a list of downregulated miRNAs in human lung epithial cells between COVID-19 patients and normal controls from a recent study [25]*.* The intersection of these upregulated miRNAs and our candidate VTMs were identified (refined VTM-target pairs).

### MiRNA/gene enrichment analysis

To better understand the functional impact of our complete VTM-target pairs, we performed miRNA over-representation analysis using the miEAA web-service (https://ccb-compute2.cs.uni-saarland.de/mieaa2/) [26]. All VTMs that are present in our complete VTM-target pairs were submitted to miEAA as a testset. Several annotations to help us interpret our results were obtained, including miRNA functional pathways (miRWalk) [27], KEGG pathways [28], target genes etc. Results were filtered to have FDR adjusted *p* value < 0.05 and a minimum interaction number of 10. Additionally, for our refined VTM-target pairs, we checked if any of these pairs have been previously reported or can be mechanistically associated with pathways related to viral infection through literature search. The VTM-target names and disease terms such as "COVID-19", "SARS-CoV-2" and "Viral infection" were submitted as keywords to google scholar to identify related studies [29].

### Single-cell RNA-sequencing data analysis

We retrieved single-cell RNA-sequencing (scRNA-seq) data on bronchoalveolar lavage fluid (BALF) samples from a recent study [30]. A total of 13 samples were obtained from the Gene Expression Omnibus (GEO) with accession numbers GSE145926 and GSM3660650. Six of them were severe COVID-19 cases, 3 of them were moderate COVID-19 cases and 4 of them were healthy controls. Data preprocessing steps such as dimensionality reduction and clustering were carefully followed as described in the original paper using R library Seurat v4.0 [31]. Original R codes and associated metadata were retrieved from https://github.com/zhangzlab/covid_balf. Cell type annotations were retrieved from the same GitHub page with file name *all.cell.annotation.meta.txt*. After cell type annotation, DEGs between severe COVID-19 cases and moderate cases for each cell types were identified using the Wilcoxon Rank-Sum test. Again, we limited the expression change of candidate genes to be between 1 and 2 folds, or equivalently a log_2_ fold change between 0 and 1 (ratio between gene expression in severe cases over gene expression in moderate cases) with Bonferroni corrected *p* value < 0.05. Potential VTMs associated with these marker genes were identified as previously described.

## Supplementary Information

**Additional file 1.** 26 miRNA families enriched in VTMs.

**Additional file 2.** 218 candidate VTM-gene pairs.

**Additional file 3.** 885 genes significantly enriched by targeting VTMs.

**Additional file 4**: miRWalk pathways identified using candidate VTMs.

**Additional file 5**: VTM-gene pairs by cell type identified from single-cell RNA-seq based analyses.

## Data Availability

The datasets analyzed during the current study are available at: https://github.com/czbiohub/covid19-transcriptomics-pathogenesis-diagnostics-results. Single-cell RNA-sequencing data were obtained from the Gene Expression Omnibus (https://www.ncbi.nlm.nih.gov/geo/) with accession numbers GSE145926 and GSM3660650. The associated R codes and metadata were obtained from https://github.com/zhangzlab/covid_balf. The candidate miRNA-gene pairs and results for enrichment analyses generated during the current study are available at Additional files 1–5. R codes used in this analysis were deposited on GitHub at: https://github.com/Chang-Li2019/COVID_Sponge.

## Abbreviations

SARS: Severe acute respiratory syndrome
COVID-19: The coronavirus disease 2019
ARDS: The Acute Respiratory Distress Syndrome
UTR: Untranslated region
scRNA-seq: Single-cell RNA-sequencing
NCBI: National Center for Biotechnology Information
VTM: Virus targeting microRNA
NP/OP: Nasopharyngeal/oropharyngeal
PCR: Polymerase chain reaction
DEG: Differentially expressed gene
FDR: False discovery rate
HGNC: HUGO Gene Nomenclature Committee
BALF: Bronchoalveolar lavage fluid
GEO: Gene Expression Omnibus
